# Retinal and brain imaging modalities for AD detection and monitoring

**DOI:** 10.1002/alz.090889

**Published:** 2025-01-09

**Authors:** Ralph N Martins, Purna Poudel, Kevin Taddei, Shaun Eslick, Shaun Frost, Eugene Hone

**Affiliations:** ^1^ Australian Alzheimer's Research Foundation, Perth, Western Australia Australia; ^2^ Macquarie University, Sydney, NSW Australia; ^3^ Edith Cowan University, Perth, Western Australia Australia; ^4^ Lions Alzheimer's Foundation, Perth, Western Australia Australia; ^5^ CSIRO, Perth, Western Australia Australia

## Abstract

**Background:**

As an extension of the central nervous system (CNS), the retina shares many similarities with the brain and can manifest signs of various neurological diseases, including Alzheimer’s disease (AD). This study investigates the spectral features of the retina to develop a classification model for the differentiation of individuals with elevated brain amyloid levels.

**Method:**

Participants (n=66) with varying brain Aβ levels, as determined by brain imaging, were non‐invasively imaged using a hyperspectral retinal camera at wavelengths of 450 to 905 nm. Multiple image features from the central view and superior view of retinas were selected and analysed to identify their variability among individuals with different brain amyloid loads. A machine learning classification model was trained to differentiate individuals with varying amyloid levels using the spectra of extracted retinal features.

**Result:**

The retinal reflectance spectra between the wavelengths of 405–585 nm exhibited a significant difference between the individuals with raised brain amyloid levels. The performance of the spectral classification model was dependent on retinal region and showed 0.778–0.857 accuracy, 0.718–0.909 sensitivity, 0.778–0.912 specificity, and 0.758–0.878 AUC.

**Conclusion:**

This study highlights the spectral variation of retinal features associated with brain amyloid load. It also demonstrates the feasibility of the retinal hyperspectral imaging technique as a potential inexpensive screening method to identify individuals in the preclinical phases of AD and as a complementary or alternative to brain imaging.

